# Healthcare-Associated Infections and Shanghai Clinicians: A Multicenter Cross-Sectional Study

**DOI:** 10.1371/journal.pone.0105838

**Published:** 2014-08-22

**Authors:** Yunfang Zhou, Dangui Zhang, Youting Chen, Sha Zhou, Shuhua Pan, Yuanchun Huang, William Ba-Thein

**Affiliations:** 1 Department of Infection Diseases, Shanghai Children's Medical Center Affiliated to Shanghai Jiao Tong University Medical College, Shanghai, P.R. China; 2 Research Center of Translational Medicine, Second Affiliated Hospital of Shantou University Medical College, Shantou, Guangdong, P.R. China; 3 Department of Clinical Microbiology, First Affiliated Hospital of Shantou University Medical College, Shantou, Guangdong, P.R. China; 4 Department of Microbiology and Immunology, Shantou University Medical College, Shantou, Guangdong, P.R. China; 5 Shantou-Oxford Clinical Research Unit, Shantou University Medical College, Shantou, Guangdong, P.R. China; University of Geneva, Switzerland

## Abstract

Literature about healthcare-associated infection (HCAI) in China is scarce. A cross-sectional anonymous survey was conducted on 647 clinicians (199 physicians and 448 nurses) from six Shanghai hospitals (grades A–C) to investigate their cognizance, knowledge, attitude, self-reported practice, and risks regarding HCAI with emphasis on precautions. The mean overall score of HCAI knowledge was 40.89±11.4 (mean±SD; range, 13∼72) out of 100 for physicians and 43.48±9.9 (10∼70) for nurses. The respondents generally received high scores in hand hygiene, HCAI core concept, and healthcare worker safety but low scores in HCAI pathogen identification and isolation precautions. There were substantial variations in the knowledge scores of various demographic groups across individual hospitals and within hospital grades (ps<0.05). Within-hospital comparisons showed that the nurses were better than physicians particularly in hand hygiene knowledge in 4 hospitals (ps<0.05). Multiple linear regression analysis showed that longer work experience was inversely and independently associated with the overall and categorical knowledge of nurses, whereas independent associations between older age or higher education and categorical knowledge were noted for physicians. The respondents' self-reported practices and adherence to standard precautions were less than satisfactory. This multi-center study reports a high level of cognizance, patchy knowledge, suboptimal adherence to infection control precautions, and self-protective attitudes among the practicing clinicians regarding HCAI, with potential safety risk to patients and healthcare providers. Providing quality learning resources, enforcing knowledge-informed practice, and promoting a healthcare safety culture are recommended as interventions. Future studies are warranted for social and behavioral aspects of healthcare safety with emphasis on infection control.

## Introduction

Infections acquired in health-care settings or healthcare-associated infections (HCAIs) are the most frequent adverse event in health-care delivery worldwide. HCAIs can occur as a part of an endemic or epidemic situation [1]. With its associated prolonged hospital stay, increased morbidity and mortality, extra financial burden, and increased microbial resistance to antimicrobials, HCAI has drawn priority attention of health authorities in many countries.

The recognized risk factors of HCAI are of patient-related (e.g., immune suppression), iatrogenic (e.g., invasive procedures), or organizational (e.g., understaffing), of which the latter two are preventable or avoidable. Since these preventable risk factors vary between and within the countries, or even within the institution, identifying local determinants of HCAI and its burden via coordinated reporting and surveillance systems at the institutional and national levels play an indispensible role in HCAI management and control.

Surveillance systems for HCAI exist in several high-income countries but are virtually nonexistent in most low- and middle-income countries [1] perhaps because the diagnosis of and reporting HCAI is complex, resource-expensive, and sometimes politically or economically sensitive. Therefore, HCAI risk assessment from surveillance on healthcare workers' perception, attitudes, behaviors, compliance, and/or perceived risks has become an alternative research strategy in developing countries [2]–[6].

National and institutional HCAI surveillance, reporting, and control systems in China are nontransparent and underdeveloped. HCAI incidence rates in some Chinese cities, which have been reported in limited literature, are comparable to or even lower than that of other countries [7]–[9] despite considerable shortcomings in the knowledge as well as observed and self-reported compliance of healthcare workers (HCW) [8], [10]. The situation of HCAIs in most hospitals across China, nonetheless, remains to be discovered.

This study aimed to fill in this information gap by investigating hospital-based physicians and nurses in Shanghai, the largest city of China, on their knowledge, attitude, practice, and risk concerning HCAI.

## Materials and Methods

### Ethics statement

The written informed consent was obtained from each participant and the study was approved by the ethics committee of A1 hospital.

### Background of participants and study sites

During June-July 2010, we conducted a cross-sectional, self-administered, anonymous survey on hospital-based physicians and nurses using the questionnaire-survey instrument that we previously used for clinical medical students [11], with slight modification in the practice section.

In China, hospitals are classified as grades A, B, and C, with the A being the highest grade, based primarily on the facility, number of beds, and clinician credentials. Participants in this study were 647 healthcare professionals (199 physicians and 448 nurses) from six hospitals, which represent specialized, general, and community hospitals in Shanghai. Two hospitals from each grade are referred herein as A1, A2, B1, B2, C1, and C2. A1 and A2 are teaching hospitals. Hospitals and participants were selected by convenience.

### Questionnaire and survey administration

The questionnaire, which was designed after the United States-Centers For Disease Control (US-CDC)'s HCAI concept and precautions, contained 53 question items with 418 possible answers (see File S1) to investigate HCW's knowledge, attitude, self-reported practice, and risks related to HCAI. The knowledge section (25 items with 272 possible answers) assessed the HCAI core concept, sources, modes of transmission, standard and isolation precautions, adjunctive measures, such as visitor management, immunoprophylaxis, and reportable infectious diseases for HCWs, and selected notable infectious diseases such as tuberculosis, *Clostridium difficile*-associated infections, and influenza. Survey questionnaires were distributed at the end of departmental academic meetings or at their work place and collected within 30 min by our study staff.

### Scoring

Only the knowledge section was scored. Correct answers were taken from the US-CDC guideline for isolation precautions 2007 [12]. A score of 1 was given to every correct question answered, no marks deducted for wrong answers, and unanswered questions were not scored. Scores were calculated as follows:

Overall score  =  (no. of correct answers ÷ the total no. of possible correct answers) ×100; Categorical score  =  (no. of correct answers in each category ÷ total no. of possible correct answers in each category) ×100.

### Data analysis

Respondents' answers were classified into knowledge, attitude, practice, and risks of HCAI and analyzed with SPSS ver.13 (SPSS, Chicago, IL). Chi-square method was used for analysis of single choice questions; T-test and one-way ANOVA method for comparing the overall scores and categorical concepts score; Kruskal-Wallis test for non-normally distributed data; and multivariate linear regression analysis to assess the factors-associated with the overall and categorical scores. Only those explanatory variables that were significantly related to the response variable were considered for inclusion in the regression model. Independent variables included in the regression model for physicians were age (continuous, in years), gender (male = 0, female = 1), and education (junior college = 0, bachelor = 1, master = 2, doctorate = 3), and for nurses were age (continuous, in years), education (junior college = 0, bachelor = 1, master = 2, doctorate = 3), position (chief = 0, associate chief = 1, nurse-in-charge = 2, registered = 3, attending = 4), and work experience (continuous, in years). All statistical tests were two-tailed, and p-value<0.05 was considered statistically significant.

## Results

### Demographics

A total of 660 questionnaires were distributed at the end of academic meetings or at the work place and collected after 30 min by our study staff, with 100% return rate; among them 647 were complete and eligible to be included in the study. Respondents comprised 56 males and 591 females (49 male physicians, 150 female physicians, 11 male nurses, and 437 female nurses), representing 19.5% (199/1021) of total physicians, 32.1% (448/1397) of total nurses, and 40.7% (11/27) of total male nurses in the participating hospitals. The majority (61.8%) of respondents and all the male nurses were from the A1 hospital (Tables 1 and 2). The majority of physicians (59.3%) were 25–35 yr old and nurses (62.6%) were ≤30 yr old. There were more physicians with a postgraduate degree in the higher-grade hospitals (45.5% in A1 and 37.5% in A2) than in other hospitals (23.8% in B1 and 0% in B2, C1, and C2). However, the B1 hospital had the largest number of nurses (28.6%) with a bachelor degree.

**Table 1:** Demographic information and HAI-knowledge overall score of physicians.

			Grade A Hospitals (n = 122)				Grade B Hospitals (n = 36)				Grade C Hospitals (n = 41)				
	Total (n = 199)		A1 (n = 90)		A2 (n = 32)		B1 (n = 21)		B2 (n = 15)		C1 (n = 25)		C2 (n = 16)		
Demo. info.	n (%)	mean±SD	n (%)	mean±SD	n (%)	mean±SD	n (%)	mean±SD	n (%)	mean±SD	n (%)	mean±SD	n (%)	mean±SD	*P* *
***Gender***															
Male	49 (24.6)	40.47±10.2	20 (22.2)	36.55±10.2	10 (31.3)	46.30±10.2^a^	0 (0)	—	5 (33.3)	38.80±8.2	10 (40.0)	43.00±10.1	4 (25.0)	41.25±6.6	0.131
Female	150 (75.4)	41.03±11.8	70 (77.8)	40.74±13.2	22 (68.7)	45.91±11.4^b^	21 (100)	38.57±11.1	10 (66.7)	40.90±9.4	15 (60.0)	42.53±9.8	12 (75.0)	36.33±7.3	0.236
***Age(yr)***															
<25	21 (10.6)	37.33±10.5	18 (20)	36.56±2.6	1 (3.1)	50.00±0	1 (4.8)	36.00±0	0 (0)	—	1 (4.0)	40.00±0	0 (0)	—	0.68
25–30	75 (37.7)	42.48±10.4	34(37.8)	43.21±11.4	13 (40.6)	48.15±9.5	12 (57.1)	37.50±9.9	6 (40)	38.00±3.8	3 (12.0)	45.00±10.4	7 (43.8)	39.71±7.1	0.121
31–35	43 (21.6)	39.30±12.0	17 (18.9)	38.12±13.1	6 (18.8)	42.67±11.9	2 (9.5)	33.00±28.3	4 (26.7)	37.75±9.9	7 (28.0)	44.43±10.2	7 (43.8)	36.86±8.2	0.746
36–40	31 (15.6)	38.94±13.3	14 (15.6)	38.00±16.8	6 (18.8)	42.17±13.0	4 (19.0)	39.25±7.7	1 (6.7)	27.00±0	6 (24.0)	39.67±8.7	0 (0)	—	0.885
>40	27 (13.6)	44.74±10.9	5 (5.6)	42.60±12.1	6 (18.8)	48.00±12.6	2 (9.5)	50.50±7.8	4 (26.7)	49.25±7.4	8 (32.0)	43.00±11.6	2 (12.5)	32.50±0.7	0.487
Missing data	2 (1.0)	31.50±10.6	2 (2.2)	31.50±10.6	0 (0)	—	0 (0)	—	0 (0)	—	0 (0)	—	0 (0)	—	/
***Education***															
Junior college	35 (17.6)	40.06±10.2	10 (11.1)	34.60±10.5	0 (0)	—	2 (9.5)	39.00±4.2	2 (13.3)	52.50±10.6	18 (72.0)	42.33±9.1	3 (18.8)	37.00±11.4	0.127
Bachelor	100 (50.3)	41.21±10.4	35 (38.9)	40.94±10.1	20 (62.5)	48.10±9.5^c^	13 (61.9)	37.23±13.4	13 (86.7)	38.31±7.2	6 (24.0)	42.33±12.3	13 (81.3)	37.00±6.5	0.017
Master	44 (22.1)	40.93±14.2	30 (33.3)	39.27±15.9	9 (28.1)	47.56±9.5	5 (23.8)	39.00±4.6	0 (0)	—	0 (0)	—	0 (0)	—	0.297
Doctor	14 (7.0)	38.14±12.7	11 (12.2)	41.00±12.5	3 (9.4)	27.67±8.0	0 (0)	—	0 (0)	—	0 (0)	—	0 (0)	—	0.11
Missing data	6 (3.0)	46.67±10.9	4 (4.4)	43.75±12.8	0 (0)	—	1 (4.8)	53.00±0	0 (0)	—	1 (4.0)	52.00±0	0 (0)	—	/
***Position/title***															
Chief	8 (4.0)	46.38±10.1	2 (2.2)	53.50±4.9	3 (9.4)	48.33±12.7	0 (0)	—	2 (13.3)	43.00±7.1	0 (0)	—	1 (6.3)	33.00±0	0.458
Assoc. chief	17 (8.5)	41.53±16.7	5 (5.6)	32.80±21.5	7 (21.9)	40.71±15.5	1 (4.8)	56.00±0	2 (13.3)	52.50±10.6	2 (8.0)	48.00±11.3	0 (0)	—	0.559
Attending	59 (29.6)	40.39±11.1	22 (24.4)	39.04±12.8	8 (25)	46.13±8.8	6 (28.6)	37.67±13.4	7 (46.7)	37.00±8.6	12 (48.0)	41.83±10.4	4 (25.0)	42.00±7.1	0.597
Resident	65 (32.7)	41.58±11.5	26 (28.9)	42.27±13.2	14 (43.8)	48.14±9.2	9 (42.9)	37.00±8.6	3 (20.0)	40.33±3.1	7 (28.0)	40.33±9.6	6 (37.5)	32.33±3.4	0.066
Intern	17 (8.5)	42.71±8.8	12 (13.3)	41.83±9.3	0 (0)	—	2 (9.5)	39.50±5.0	0 (0)	—	2 (8.0)	51.50±7.8	1 (6.3)	42.00±0	0.532
Others	6 (3.0)	31.00±9.6	4 (4.4)	27.75±7.9	0 (0)	—	0 (0)	—	0 (0)	—	0 (0)	—	2(12.5)	37.50±12.0	0.285
Missing data	27 (13.6)	39.37±7.8	19 (21.1)	39.00±10.7	0 (0)	—	3 (14.3)	38.67±8.5	1 (6.7)	32.00±0	2 (8.0)	42.50±9.2	2 (12.5)	44.50±7.8	/
***Work experience (yr)***															
<5	47 (23.6)	41.72±11.5	20 (22.2)	39.70±13.6	12 (37.5)	49.83±9.0^d^	4 (19.0)	35.25±5.1	3 (20.0)	35.33±2.9	3 (12.0)	45.67±9.8	5 (31.3)	37.00±4.0	0.026
5∼10	49 (24.6)	38.39±10.3	16 (17.8)	39.75±11.3	8 (25)	38.63±6.8	8 (38.1)	34.00±14.2	7 (46.7)	39.00±7.3	4 (16.0)	39.25±12.7	6 (37.5)	39.00±10.0	0.89
11∼15	25 (12.6)	42.8±11.1	11 (12.2)	40.45±14.2	3 (9.4)	51.67±4.6	2 (9.5)	42.50±14.8	0 (0)	—	7 (28.0)	44.86±5.3	2 (12.5)	35.50±9.2	0.368
16∼20	23 (11.6)	40.96±11.3	6 (6.7)	39.00±11.5	6 (18.8)	40.67±15.9	3 (14.3)	44.67±5.5	2 (13.3)	35.50±12.0	6 (24.0)	43.17±10.3	0 (0)	—	0.9
>20	15 (7.5)	46.13±11.6	1 (1.1)	50.00±0	3 (9.4)	55.67±2.5	1 (4.8)	56.00±0	3 (20.0)	51.00±7.9	5 (20.0)	40.20±13.7	2 (12.5)	32.50±0.7	0.169
Missing data	40 (20.1)	39.64±12.7	36 (40.0)	39.56±13.2	0 (0)	—	3 (14.3)	40.67±4.0	0 (0)	—	0 (0)	—	1 (6.3)		/
*Total*	199 (100)	40.89±11.4	90 (100)	39.81±12.7	32 (100)	46.03±10.9	21 (100)	38.57±11.1	15 (100)	40.20±8.8	25 (100)	42.72±9.7	16 (100)	37.56±7.3	0.068

Overall score  =  (no. of correct answers ÷ the total no. of possible correct answers) ×100.

*vs. the same demographic group across hospitals, analyzed by One-way ANOVA.

a∼d vs. the same demographic group of another hospital in the same hospital grade analyzed by T test, P<0.05.

**Table 2:** Demographic information and HAI-knowledge overall score of nurses.

			Grade A Hospitals (n = 356)				Grade B Hospitals (n = 43)				Grade C Hospitals (n = 49)				
	Total (n = 448)		A1 (n = 310)		A2 (n = 46)		B1 (n = 28)		B2 (n = 15)		C1 (n = 30)		C2 (n = 19)		
Demo. info.	n (%)	mean±SD	n (%)	mean±SD	n (%)	mean±SD	n (%)	mean±SD	n (%)	mean±SD	n (%)	mean±SD	n (%)	mean±SD	*P* *
***Gender***															
Male	11 (2.5)	47.50±13.7	11 (3.5)	47.45±13.7	0 (0)	—	0 (0)	—	0 (0)	—	0 (0)	—	0 (0)	—	/
Female	437 (97.5)	42.26±10.9	299 (96.5)	45.67±9.1	46 (100)	37.48±11.4 ^a^	28 (100)	43.07±7.3	15 (100)	45.00±7.1	30 (100)	32.90±7.1	19 (100)	37.32±4.2	<0.0001
***Age(yr)***															
<25	149 (33.3)	42.46±9.8	121 (39.0)	44.00±9.5	10 (21.7)	31.20±6.9 ^b^	5 (17.9)	46.80±10.8	4 (26.7)	40.75±0.5	3 (10.0)	27.33±5.7	6 (31.6)	37.83±3.7 ^c^	<0.0001
25–30	132 (29.5)	45.34±8.9	103 (33.2)	47.09±8.2	14 (30.4)	41.93±8.5 ^d^	8 (28.6)	41.00±5.2	0 (0)	—	4 (13.3)	26.75±10.6	3 (15.8)	37.67±0.6	<0.0001
31–35	89 (19.9)	44.46±9.1	52 (16.8)	47.73±8.1	7 (15.2)	41.57±10.7	5 (17.9)	40.80±7.1	6 (40.0)	49.33±7.2	14 (46.7)	34.71±6.4	5 (26.3)	39.60±3.2	<0.0001
36–40	39 (8.7)	43.03±10.6	20 (6.5)	47.05±9.6	7 (15.2)	37.00±14.3 ^e^	4 (14.3)	45.75±6.2	3 (20.0)	41.33±5.5	1 (3.3)	36.00±0	4 (21.1)	33.75±6.6	0.101
>40	35 (7.8)	38.29±12.8	10 (3.2)	40.40±17.3	8 (17.4)	34.38±15.7 ^f^	6 (21.4)	42.83±7.7	2 (13.3)	46.00±12.7	8 (26.7)	34.50±5.4	1 (5.3)	36.00±0	0.696
Missing data	4 (0.9)	40.50±12.7	4 (1.3)	40.50±12.7	0 (0)	—	0 (0)	—	0 (0)	—	0 (0)	—	0 (0)	—	/
***Education***															
Vocational school	102 (22.8)	42.52±10.6	61 (19.7)	46.93±9.8	18 (39.1)	34.61±11.3 ^g^	6 (21.4)	39.50±3.3	2 (13.3)	39.00±2.8	5 (16.7)	33.00±8.5	10 (52.6)	37.10±1.4	0.003
Junior college	232 (51.8)	43.04±10.1	162 (52.3)	44.87±9.8	24 (52.2)	38.25±11.3 ^h^	12 (42.9)	42.50±6.4	12 (80.0)	44.75±6.1	17 (56.7)	32.41±7.8	5 (26.3)	40.20±4.4 ^i^	0.169
Bachelor	83 (18.1)	45.83±8.4	64 (20.6)	46.59±8.0	4 (8.7)	45.75±10.3	8 (28.6)	45.63±10.2	1 (6.7)	60.0±0	5 (16.7)	36.40±4.7	1 (5.3)	38.0±0	0.28
Missing data	31 (6.9)	41.94±9.1	23 (7.4)	44.57±7.3	0 (0)	—	2 (7.1)	47.00±7.1	0 (0)	—	3 (10.0)	28.00±3.0	3 (15.8)	32.33±9.0	/
***Position/title***															
Chief	1 (0.2)	34.00±0	0 (0)	—	0 (0)	—	0 (0)	—	0 (0)	—	0 (0)	—	1 (5.3)	34.00±0	/
Assoc. chief	50 (13.4)	41.02±9.9	21 (6.8)	46.62±7.9	12 (26.1)	36.33±14.6 ^j^	8 (28.6)	42.75±8.1	4 (26.7)	40.50±5.1	5 (16.7)	34.20±3.8	0 (0)	—	0.001
Nurse-in-charge	156 (33.9)	45.59±9.4	105 (33.9)	47.70±8.9	9 (19.6)	43.00±9.8	11 (39.3)	43.36±5.6	6 (40.0)	51.33±6.8 ^k^	15 (50.0)	34.73±7.1	10 (52.6)	37.10±5.2	0.291
Registered	204 (44.0)	43.25±9.6	159 (51.3)	44.76±9.1	20 (43.5)	37.20±10.4 ^l^	6 (21.4)	40.33±2.7	5 (33.3)	41.00±0.7	8 (26.7)	30.25±7.9	6 (31.6)	37.83±3.7	0.013
Attending	7 (1.3)	37.33±15.7	0 (0)	—	4 (8.7)	29.00±6.2	2 (7.1)	54.00±17.0	0 (0)	—	0 (0)	—	1 (5.3)	36.00±0	0.039
Missing data	30 (6.7)	39.33±10.6	25 (8.1)	40.56±10.6	1 (2.2)	40.00±0	1 (3.6)	41.00±0	0 (0)	—	2 (6.7)	24.50±7.8	1 (5.3)	36.00±0	/
***Work experience (yr)***															
<5	147 (32.8)	43.97±9.4	113 (36.5)	46.38±8.2	15 (32.6)	33.13±7.5 ^m^	8 (28.6)	42.88±10.0	3 (20.0)	40.67±0.6	4 (13.3)	28.75±10.4	4 (21.1)	36.50±1.3	<0.0001
5∼10	96 (21.4)	45.76±9.6	72 (23.2)	46.94±9.7	10 (21.7)	45.50±8.0	5 (17.9)	44.20±3.3	2 (13.3)	47.50±7.8	2 (6.7)	24.00±7.1	5 (26.3)	38.80±9.6 ^n^	0.012
11∼15	72 (16.1)	44.78±8.3	45 (14.5)	47.33±7.6	4 (8.7)	43.00±2.7	5 (17.9)	41.60±7.9	5 (33.3)	48.40±8.0	10 (33.3)	35.20±6.7	3 (15.8)	40.00±4.4	<0.0001
16∼20	40 (8.9)	40.85±12.8	21 (6.8)	48.33±7.9	9 (19.6)	25.33±12.6 ^o^	3 (10.7)	44.00±8.2	2 (13.3)	44.00±4.2	1 (3.3)	36.00±0	4 (21.1)	33.75±6.6	<0.0001
>20	43 (9.6)	37.05±12.2	12 (3.9)	43.42±15.2	7 (15.2)	22.57±3.4 ^p^	7 (25.0)	44.86±8.1	3 (20.0)	42.67±10.7	13 (43.3)	33.54±5.7	1 (5.3)	36.00±0	0.001
Missing data	50 (11.2)	37.58±9.5	47 (15.2)	37.91±9.5	1 (2.2)	23.00±0	0 (0)	—	0 (0)	—	0 (0)	—	2 (10.5)	37.00±1.4	/
*Total*	448 (100)	43.48±9.9	310 (100)	45.74±9.3	46 (100)	37.48±11.4	28 (100)	43.07±7.3	15 (100)	45.00±7.1	30 (100)	32.90±7.1	19 (100)	37.32±4.2	<0.0001

Overall score  =  (no. of correct answers ÷ the total no. of possible correct answers) ×100.

*vs. the same demographic group across hospitals, analyzed by One-way ANOVA.

a∼p vs. the same demographic group of another hospital in the same hospital grade, analyzed by T test, P<0.05.

A majority of respondents were accounted for by residents and attending physicians (62.3%) or registered nurses and nurses in charge (80.3%); 56.3% of physicians and 56.0% of nurses were in practice for ≥5 years) (Tables 1 and 2).

### Cognizance and knowledge

Almost all physicians and nurses (>90%) acknowledged the cases of HCAIs in their hospitals, with respiratory tract infections being the most commonly seen, followed by surgical site infections, gastrointestinal infections, urinary tract infections, or bloodstream infections.

#### Overall knowledge

The mean overall score of HCAI-knowledge was 40.89±11.4 (range 13∼72) out of 100 for physicians and 43.48±9.9 (10∼70) for nurses (Tables 1 and 2), with significant differences between them in the hospitals A1 (39.81±12.7 [95% CI, 37.2∼42.5] vs. 45.74±9.3 [44.7∼46.8], p<0.001), A2 (46.03±10.9 [42.1∼50.0] vs. 37.48±11.4 [34.1∼40.9], p = 0.001), and C1 (42.72±9.7 [38.7∼46.7] vs. 32.90±7.1 [30.3∼35.5], p<0.001) (data not shown). There were only 18 (2.8%) who achieved the arbitrary passing score of ≥60, including one physician and one nurse scoring 72 and 70, respectively (Table 3).

**Table 3:** Number (%) of physicians and nurses with their overall scores in HCAI knowledge.

Overall score	Physicians (n = 199)	Nurses (n = 448)
70–72	1 (0.50)	1 (0.22)
60–69	7 (3.52)	9 (2.01)
50–59	43 (21.61)	68 (15.18)
40–49	58 (29.15)	192 (42.86)
30–39	56 (28.14)	137 (30.58)
20–29	29 (14.57)	29 (6.47)
0–19	5 (2.51)	12 (2.68)

Note: The arbitrary passing score is ≥60.

Among the physicians, those in the A2 hospital received the highest average overall score (46.03±10.9), while their counterparts in the C2 received the lowest (37.56±7.3), with a statistically significant difference (p = 0.015). On the other hand, nurses in the A1 hospital scored highest and those in the C1 scored the lowest (45.74±9.3 vs. 32.90±7.1, p<0.001); In general, the nurses of grade A and B hospitals performed significantly better than those of grade C hospitals (ps<0.001).

We observed substantial variations in the knowledge scores of various demographic groups across individual hospitals and within hospital grade. In cross-hospital comparisons, differences were found in two demographic groups (education and work experience) for physicians (ps 0.017 and 0.026, table 1) and all demographic groups (gender, age, education, position, and work experience) for nurses (ps<0.001∼0.039, table 2). Within-hospital grade comparisons for physicians showed that the gender, education, and work experience groups of A2 hospital were better than their demographic peers in the A1 hospital (ps<0.05, table 1). Whereas for nurses, all demographic groups of A1 hospital; the position/title group of B2 hospital; the age, education, and work experience groups of C2 hospital performed better than their demographic peers (ps<0.05, table 2).

#### Categorical knowledge

When their knowledge scores were broken down into seven categories (Table 4), both physicians and nurses generally received high scores in HCAI core concept, hand hygiene, and HCW safety but low scores in HCAI pathogen identification, isolation precautions, and personal protective equipment (PPE) use. Cross-hospital comparisons showed significant differences in the categorical knowledge scores among the physicians (ps<0.0001∼0.034) and the nurses (ps<0.0001). In within-hospital comparisons, the physicians in the A2, C1, and C2 hospitals scored better than did their fellow nurses in some categories (ps<0.05) (Table 4). However, the nurses performed better than the physicians in all 7 categories (ps<0.05) in the A1 hospital. They also outperformed physicians in hand hygiene in the B2, C1, and C2 hospitals (ps<0.05).

**Table 4:** The categorical HAI-knowledge scores of physicians and nurses from 6 hospitals.^a^

	Physicians (n = 199)							Nurses (n = 448)						
Knowledge items	A1 (n = 90)	A2 (n = 32)	B1 (n = 21)	B2 (n = 15)	C1 (n = 25)	C2 (n = 16)	P^b^	A1 (n = 310)	A2 (n = 46)	B1 (n = 28)	B2 (n = 15)	C1 (n = 30)	C2 (n = 19)	P^b^
Core concept	66.67±47.4	75.00±44.0	85.71±35.9	86.67±35.2	68.00±47.6^c^	62.50±50.0	0.333	87.74±32.9^d^	80.43±40.1	85.71±35.6	66.67±48.8	36.67±49.0	73.68±45.2	<0.0001
HCA-pathogens	26.67±14.4	35.36±18.8^c^	20.80±17.3	17.90±13.0	27.79±13.5^c^	16.78±13.5	<0.0001	34.92±15.0^d^	25.52±18.1	23.31±15.3	22.81±10.8	18.60±17.5	19.11±9.2^d^	<0.0001
HCAI Source	58.33±24.2	60.49±19.7^c^	46.94±19.1	49.76±14.6	65.43±16.0^c^	52.23±16.1	0.018	64.70±17.3^d^	49.69±17.5	62.12±10.7^d^	68.10±17.4^d^	44.05±12.4	51.50±12.5	<0.0001
Hand hygiene	68.49±20.5	78.35±20.3	70.41±21.9	62.38±14.7	67.43±18.6	71.87±13.4	0.004	86.47±11.3^d^	77.17±18.5	78.06±9.5	88.09±8.0^d^	77.86±12.4^d^	93.61±5.3^d^	<0.0001
PPE	53.38±19.3	58.0±15.6^c^	54.37±13.3	55.28±9.5	59.00±16.0	43.23±19.9	0.034	59.97±12.4^d^	44.57±21.9	58.19±14.1	60.28±9.2	54.58±11.9	39.69±16.8	<0.0001
HCW safety	65.56±29.5	66.25±31.5^c^	75.24±20.9	80.00±22.7	76.00±23.8	76.25±23.4	0.306	87.10±21.3^d^	43.04±27.6	65.00±24.7	81.33±26.7	79.33±25.5	77.89±11.3	<0.0001
Isolation precautions	45.56±19.9	49.22±19.3^c^	48.81±23.3	56.67±9.6	46.33±14.8	55.73±16.0^c^	0.085	50.46±16.8^d^	36.96±15.9	42.26±13.8	48.89±14.4	43.33±11.2	36.40±6.3	<0.0001

PPE, personal protective equipment; HCW, healthcare worker.

a (no. of correct answers in each category/total no. of possible correct answers in each category) ×100.

b non-normally distributed data, analyzed by *Kruskal Wallis* method.

c Physicians are better than nurses in the same hospital for the same categorical item, analyzed by independent T-test, p<0.05.

d Nurses are better than physicians in the same hospital for the same categorical item, analyzed by independent T-test, p<0.05.

#### Factors associated with HCAI knowledge

The multivariate linear regression analysis (Table 5) showed that work experience of the nurses was inversely and independently associated with their overall knowledge and categorical knowledge (except hand hygiene). On the other hand, for the physicians, there were independent associations between older age and knowledge about core concept and HCW safety, and between higher education level and knowledge of pathogen identification and hand hygiene.

**Table 5:** Multivariate linear regression analysis of HAI knowledge-associated factors in physicians and nurses (n = 647).

				Categorical knowledge																				
	Overall knowledge			Core concept			HCA-Pathogens			HCAI Sources			Hand hygiene			PPE			HCW safety			Isolation precautions		
	Coeff.	SE	*P*	Coeff.	SE	*P*	Coeff.	SE	*P*	Coeff.	SE	*P*	Coeff.	SE	*P*	Coeff.	SE	*P*	Coeff.	SE	*P*	Coeff.	SE	*P*
**Physicians (n = 199)**																								
Older age	0.147	0.106	0.168	0.889	0.156	0.035	10207	0.144	0.153	0.002	0.193	0.993	0.302	0.184	0.102	0.173	0.163	0.289	0.552	0.256	0.032	0.186	0.177	0.294
Being female	0.952	1.952	0.626	5.908	0.056	0.444	−4.124	2.652	0.122	−5.54	3.556	0.121	−1.747	3.39	0.607	−4.714	2.993	0.117	−1.971	4.712	0.676	0.764	3.256	0.815
Higher education	−0.174	1.024	0.865	0.363	4.041	0.929	2.967	1.392	0.034	0.913	1.867	0.626	3.771	1.78	0.035	1.096	1.571	0.486	−2.624	2.474	0.29	0.729	1.709	0.67
**Nurses (n = 448)**																								
Older age	0.103	0.085	0.225	0.372	0.349	0.287	−0.069	0.138	0.619	0.185	0.152	0.225	−0.108	0.119	0.365	−0.197	0.134	0.142	−0.123	0.243	0.612	0.108	0.154	0.483
Higher education	1.361	0.774	0.079	1.976	3.19	0.536	1.168	1.261	0.355	2.312	1.394	0.098	−1.499	1.089	0.169	0.076	1.227	0.951	−2.179	2.218	0.327	2.2	1.407	0.119
Higher position	1.269	1.364	0.353	4.995	5.625	0.375	−2.173	2.223	0.329	4.888	2.458	0.048	−1.379	1.919	0.473	−2.881	2.163	0.184	−2.32	3.911	0.553	1.416	2.481	0.569
Longer work experience	−0.52	0.094	<0.001	−1.663	0.389	<0.001	−1.02	0.154	<0.001	−0.726	0.17	<0.001	−0.199	0.133	0.135	−0.504	0.149	0.001	−0.743	0.27	0.006	−0.499	0.172	0.004

HCAI, healthcare-associated infection; PPE, personal protective equipment; HCW, healthcare worker.

### Practice

The practice section of questionnaire collected self-reported practices on hand hygiene, use and care of PPE and clinician's accessories (including white coat, stethoscope, and medical pocket watch), and handling of medical waste.

#### Hand hygiene

The majority of physicians (59.3%) and nurses (82.6%) reportedly washed their hands with running water and hand-washing liquid. Alcohol hand rub, which is available in all participating hospitals, was used by 30.0% of physicians and 50.9% of nurses. Most physicians (77.9%) and nurses (96.0%) acknowledged that hand hygiene is the single most effective measure to prevent HCAI. They performed hand hygiene correctly in general, yet nearly half of the physicians (46.7%) did not practice hand hygiene between two different procedures on the same patients; and some physicians (39.2%) and nurses (31.5%) did not wash hands after using the computers and desks in the staff station.

#### PPE

Reported PPE use by most physicians and nurses was generally acceptable. For instance, when performing lumber puncture, 83.8% and 84.9% of physicians or 89.5% and 87.7% of nurses respectively wore mask and gloves. Nonetheless, self-protective attitudes were noted from indiscriminate use of medical utility gloves in performing physical examination, making clinical rounds, using computers, and/or prescribing drugs, which was admitted by 53.3% of physicians and 64.6% of nurses.

#### Clinician's accessories

Their uniforms as a potential source of HCAI was understood by 71.4% of physicians and 58.0% of nurses; however, less than half of them laundered twice or once per week. Only clinicians from intensive care units (i.e., 5% of the respondents) laundered their white coats/nurse uniforms daily as required by hospital policy. Nearly half of the respondents did not know that stethoscopes and medical pocket watches are also a source of infections. Around 60% of nurses proclaimed that they disinfect their stethoscopes and pocket watches after use on each patient; in comparison, only 29.1% of physicians did so with their stethoscopes (P<0.001).

#### Medical waste management

Some physicians (19.6%, 39/199) and nurses (13.8%, 62/448) did not dispose medical wastes into the assigned waste bins; of note, 50% (23/46) of the A2 nurses had no idea where to dispose.

All these self-reported practices and adherence to standard precautions were less than satisfactory, compromising safety environment.

### Attitudes towards safety

Almost all respondents acknowledged that patients were the most important source of HCAI and accordingly 71.4% of physicians and 82.1% of nurses considered all patients potentially contagious. Despite that 87.4% of physicians and 91.7% of nurses regarded all unsterile needles and sharps as contaminated, 47.2% and 71.4%, respectively, of them claimed to have sustained used-needle stick injuries. Adherence to the standard precautions of both physicians and nurses was not only suboptimal but also apparently skewed towards self-protection, less towards patient care. A noteworthy example is that while 88.4% of physicians and 91.0% of nurses would wear a mask themselves, just 26.1% and 39.5% of them would put a mask on patients when transporting them for a medical procedure such as radiotherapy.

Another example is of reportable infectious diseases. A large proportion of the respondents (up to 79.9%) would not report if they had contracted highly contagious herpes zoster, influenza, or acute hemorrhagic conjunctivitis.

## Discussion

Here we report the first HCAI related investigation with hospital-based physicians and nurses in Shanghai representing three different tiers of hospitals in Chinese healthcare system. Despite increased awareness and tighter hospital infection control measures in recent years, our survey instrument enabled us to identify obvious safety concerns for patients and healthcare providers attributable to the shortcomings in the clinicians' knowledge and practices concerning HCAI.

### HCAI incidence

HCAI incidence rate in 13 grade A hospitals in China was reported as around 4% in 2006-7 [8]. A similar incidence rate has been documented in one of the hospitals under study for 2006-2012 (unpublished data). Although we do not know the HCAI incidence in other participating hospitals, high level of HCAI awareness among the respondents suggests the gravity of HCAI situation in their respective hospitals.

### Knowledge deficit

We have previously reported that Chinese medical students have substantially limited knowledge in HCAI mainly due to deficient learning resources [11]. We found similarities between our previous study and this study. The mean knowledge levels of the clinicians are unexpectedly lower than that of the students (40.89±11.4 for physicians and 43.48±9.9 for nurses vs. 52.54±0.45 for students, using the same questionnaire). Similar to the students, the clinicians also exhibited patchy knowledge as evident from their high scores in some categorical items, such as hand hygiene and HCW safety, and very low scores in knowledge about HCAI pathogens. Given that almost all clinicians (97.2%) scored below the arbitrary passing score of 60, knowledge deficit problem concerns all clinicians regardless of their demographic characteristics.

There are several possible explanations for this. As described previously [11], the curricula across Chinese medical and nursing schools are rather uniform, with little, if any, emphasis on HCAI. Besides, the related theoretical (taught) knowledge from the school is neither revisited nor reinforced under clinical settings in the internship years, in National Medical Licensing Examination (NMLE), or in clinical practice [11], therefore allowing theoretical knowledge to wane over time. Whereas the practical knowledge, the precautions in particular, is not acquired further through professional experience as reported in a Swiss study [13], leaving our respondents with knowledge gaps in developing concepts of infection control. This is supported by the regression model (Table 5) where nurses' knowledge was inversely influenced by their work experience.

On the other hand, when adherence to the precautions is not reinforced or continuing medical educational (CME) opportunities are not provided, which is the case in some participating hospitals in this study, clinicians incline to acquire knowledge-on-demand that is restricted to their work assignment only. That is what we observed as patchy knowledge. One representative example is that patients with suspected tuberculosis are directly referred to dedicated hospitals; as a result, only 9.9% of the respondents knew of *Mycobacterium tuberculosis* as an HCAI agent. Also, norovirus whose diagnosis is not available locally was known to less than 4% of the respondents. Their patchy knowledge was also presented as knowledge disparity, which is exemplified by substantial knowledge variations among the physicians especially of grade A hospitals and the nurses of all hospital grades.

Higher education background, which is one of the recruitment criteria in higher-grade Chinese hospitals, had no positive influence on their knowledge. The clinicians, especially the physicians, in two grade A hospitals were no better than those in the grade B and C hospitals in the practical knowledge especially precautions. Physicians with a Ph.D. even scored lowest in the overall knowledge (38.14±12.7).

### Suboptimal practice

We noted two problems with the practices of the clinicians: practice without adequate knowledge and poor knowledge translation. Their desirable self-reported practices were not matched with their poor knowledge scores (e.g., PPE use). In addition, lack of comprehensive understanding of hand hygiene concept indicated by their hand hygiene practices altogether illustrates “practice without adequate knowledge” that can jeopardize the safety of patients and self. Therefore, knowledge-informed practice must be promoted in the intervention.

On the other hand, good knowledge and positive attitude of the physicians was not translated into good hand hygiene compliance. As described previously in a British study [14], we also recognized the existence of a local clinical environment, where a strong hierarchical culture and practicing etiquettes endorsed by the seniors within the clinical groups, which might have played an important role in the reported suboptimal adherence to the precautions in this study. Under this circumstance, good knowledge will not assure good practice. Given that institutional behavior generally reflects leadership behavior, in addition to knowledge and practice, understanding the practicing behavior of clinicians (principally the senior opinion leaders) is of tantamount importance in designing an effective intervention.

Clinician's suboptimal adherence to infection control precautions has been well-documented [10], [13], [15]–[17], with the main given or interpreted reasons being lack of knowledge, lack of time, lack of resources, high work load, forgetfulness, and interference with the patient care. The most significant interpreted reasons standing out from our investigations with medical students [11] and clinicians in this study are knowledge deficit from lack of learning resources and undeveloped healthcare safety culture as discussed hereafter.

### Deficient learning resources

Modern clinical practice is strongly influenced by practice guidelines. Chinese hospitals are required to keep abreast with the current infection control measures. But the national guidelines in China [18], on which all institutional guidelines are based, are not on par with the existing scientific evidence and thus not the best references. As an example, the institutional guidelines for precautions in the participating hospitals have not been updated since 2000. This could be one of the major reasons for their poor performance against our survey, which is based on the US-CDC guidelines published in 2007. The same reason may apply for our finding that merely 13.07% of physicians and 3.35% of nurses knew of *Clostridium difficile* that is not included in the guidelines.

Language barrier in fact plays a central role in discouraging clinicians to review the current scientific literature including international practice guidelines. Thus, at least revising Chinese institutional guidelines in line with well-respected international guidelines is essential.

### Undeveloped healthcare safety culture

Healthcare safety, the cornerstone of medical practice and healthcare delivery, is concerned with HCW safety and patient safety. Both elements were found compromised in this study.

Compared to physicians, nurses had higher HCAI risk because they usually involve in high-risk tasks from close contact with patients, as evident from higher needle stick injuries in nurses (P<0.001). Surprisingly, no respondents in the study reacted correctly in response to accidental exposures to contaminated body fluid through intact and non-intact skin or mucous membrane. Besides, immunoprophylaxis is not mandatory under the hospital guidelines. Patient safety as well did not receive due priority in this study, which is illustrated by the respondents' self-protective attitudes along with suboptimal adherence to the precautions.

Of note, clinicians (especially physicians) casually consider HCAI as a specialty belonging to infection specialists who should be responsible for management and prevention of HCAI, thereby getting away from responsible conduct and liability. There are clinical audits (with infection control as top priority) at the national, city, and hospital levels, but compliance to the practice guidelines is usually higher during the audit period to avoid possible punishments such as hospital downgrading (unpublished personal communication with infection control personnel in the participating hospitals).

Without good awareness of these apparent risks to patients and self, the majority of physicians (72.3%) and nurses (81.7%) still expressed a positive attitude towards the infection control measures and practices in their hospitals.

These findings strongly suggest an undeveloped nature of healthcare safety culture in the participating hospitals. Consistent with this, impediments in implementing patient safety culture have been communicated in a recent study in Beijing [19]. Raising awareness and promoting a culture of healthcare safety, especially patient safety, should be the groundwork towards HCAI intervention.

### Physicians vs. Nurses

There are numerous studies describing the HCAI knowledge, attitudes, and practice of physicians and nurses practicing in various disciplines, with conflicting findings [4], [20], [21]. Our knowledge assessment showed nurses were generally better than physicians particularly in hand hygiene, perhaps because nursing job is more patient-oriented and compliance demanding. In fact, nurses reportedly have better compliance with the guidelines in an Italian study [2]. Education-on-job might have also played an important role in this regard, because the nurses in the A1 hospital, who outperformed their fellow physicians in all HCAI knowledge categories (Table 4), were regular attendees of the institutional CME activities (unpublished personal communication). Physician-focused intervention may therefore be considered as they play important roles as opinion leaders, decision makers, and role models in clinical environment.

## Recommendations

We propose three key aspects as general intervention measures for all participating hospitals: implementing quality information resource, enforcing knowledge-informed practice, and promoting healthcare safety culture.

Being the single most important information resource, the institutional guidelines on infection control should be upgraded in line with the current scientific evidence such as the US CDC standards that are accepted globally. However, having all HCWs required to comply with the practice guidelines inclusive of all aspects of infection control and prevention could be a great challenge or even counterproductive especially for busy clinicians. Therefore, our practical recommendation is to reinforce hospital-wide standard precautions in conformity with the local clinical culture, followed further for specific safety precautions against unusual circumstances, by providing minimally targeted interventions such as job-based or deficiency-based education and training to specific groups or departments. For example, targeted training on PPE for the C2 clinicians, HCW safety for the A2 nurses, or isolation precautions for all clinicians, or theoretical knowledge enrichment for long-serving senior nurses should be considered. Attitude modification, i.e., transforming self-protective attitudes into patient-safety attitudes, will only be achievable by promoting a culture of healthcare safety in the participating hospitals.

Short training courses, with the above-mentioned three key issues in focus, delivered through lectures, discussion, and demonstration, or online self-paced learning and training for busy clinicians, are recommended.

After the findings from this study, the infection control team in the A1 hospital has already initiated interventions through lectures, an online self-training course, and activities on special days, targeting HCWs, patients and their relatives, and volunteers. Post-intervention assessments are to be undertaken in due course.

## Study Limitations

Shanghai is one of the 17 nationally designated cities for healthcare reform initiated in 2009 [22]. The participating hospitals and participants were selected by convenience. In addition, there could be biases in self-reported surveys, especially aspects of behaviors and practices, which may lead to overestimation of their practices. Therefore, our report may not closely reflect the real situation in the participating hospitals or hospitals elsewhere in China. The missing data in demography might have affected our data analysis to some extent, but which should not change our interpretation. The inferences drawn from the self-reported practices could be different if direct observation was made.

## Conclusions

This multi-center study reports high level of cognizance, patchy knowledge, suboptimal adherence to infection control precautions, and self-protective attitudes among the practicing clinicians regarding HCAI, with potential safety risk to patients and healthcare providers. Providing quality learning resources, enforcing knowledge-informed practices, and promoting a healthcare safety culture with strong government commitment and support are recommended as interventions. Future studies are warranted for social and behavioral aspects of healthcare safety with emphasis on infection control.

## Supporting Information

File S1 HCAI questionnaire.
(PDF)
