# Supplementary material for: Healthcare-Associated Infections and Shanghai Clinicians: A Multicenter Cross-Sectional Study
Source: PLoS One. 2014 Aug 22;9(8):e105838. doi: 10.1371/journal.pone.0105838 (PMC4141815; doi:10.1371/journal.pone.0105838)
Supplement: File S1 — HCAI questionnaire. (PDF) [file pone.0105838.s001.pdf]

Questionnaires for healthcare-associated Infections (Physicians & Nurses)

Healthcare-associated infections (HCAIs) are infections acquired during the course of receiving treatment for other conditions within a healthcare facility.

Note: **SC**, single-choice; **MC**, multiple-choice; **PO**, Physicians only; **NO**, Nurses only.

--KNOWLEDGE SECTION---

1. Are you aware of HCAIs in your hospital? YES ☐, NO ☐

2. Which of the following HCAIs are commonly seen in your hospital? **MC**

   ☐ A. Urinary tract infections (UTI)
   ☐ B. Surgical wound infections
   ☐ C. Respiratory tract infections (RTI)
   ☐ D. Bloodstream infections (i.e., bacteremia and/or septicemia)
   ☐ E. Gastrointestinal tract infections (GITI)
   ☐ F. Skin infections
   ☐ G. Others; please specify:

3. Which infectious agents are commonly implicated in HCAIs? **MC**

   ☐ A. Methicillin-resistant *Staphylococcus aureus* (MRSA)
   ☐ B. Vancomycin-resistant enterococcus (VRE)
   ☐ C. *Pseudomonas aeruginosa*
   ☐ D. *Klebsiella pnemoniae*
   ☐ E. *Mycoplasma pneumoniae*
   ☐ F. *Streptococcus pneumoniae*
   ☐ G. *Mycobacterium tuberculosis*
   ☐ H. *Eschericheria coli*
   ☐ I. *Clostridium difficile*
   ☐ J. *Bordetella pertussis*
   ☐ K. *Acinetobacter baumannii*
   ☐ L. *Stenotrophomonas maltophilia*
   ☐ M. *Haemophilus influenzae*
   ☐ N. *Candida albicans*
   ☐ O. Rotavirus
   ☐ P. Norovirus (Norwalk virus)
   ☐ Q. Influenza virus
   ☐ R. Hepatitis A virus (HAV)
   ☐ S. Respiratory syncytial virus (RSV)
   ☐ T. Not identified

4. Can you name at least 5 pathogens most commonly isolated in your hospital? **PO**

   ____

5. Which population is the <u>most susceptible</u> to HCAIs? **SC**

   ☐ A. Inpatients
   ☐ B. Outpatients
   ☐ C. Doctors (including medical students and interns)
   ☐ D. Nurses
   ☐ E. Janitors
   ☐ F. Visitors/care givers

☐ G. Cleaner
☐ H. Animal (rat)

6. Which is the most important source/reservoir of HCAIs? **SC**

☐ A. Inpatients
☐ B. Outpatients
☐ C. Doctors (including medical students and interns)
☐ D. Nurses
☐ E. Caregivers or Attendants
☐ F. Visitors
☐ G. Cleaners
☐ H. Animals such as rats

7. Which of the following behavior(s) can spread infectious organisms? **MC**

☐ A. Coughing
☐ B. Spitting
☐ C. Talking
☐ D. Laughing
☐ E. Sneezing
☐ F. Hand shaking
☐ G. Hugging
☐ H. Kissing
☐ I. Sharing a drinking straw
☐ J. Reusing non-disposable chopsticks

8. To prevent HCAIs, you should report to your health department when you have contracted: **MC**

☐ A. Hepatitis B (HBsAg +)
☐ B. Hepatitis A
☐ C. HIV
☐ D. Herpes zoster (shingles)
☐ E. Influenza
☐ F. Food poisoning
☐ G. Acute viral (hemorrhagic) conjunctivitis
☐ H. Malaria

9. When a patient with pulmonary tuberculosis can be removed from the isolation room? **SC**

☐ A. After patient's signs and symptoms have disappeared
☐ B. When chest X-rays become negative
☐ C. After 3 consecutive negative sputum smear
☐ D. After 3 consecutive negative sputum culture

10. Which is the single most effective method to prevent HCAIs? **SC**

☐ A. Hand washing properly
☐ B. Wearing caps, masks, and shoe covers
☐ C. Regular vaccination of healthcare workers
☐ D. Isolation (cohorting) of infected/colonized patients
☐ E. Cohorting staff (assignment of staff to a cohort of patients)
☐ F. Prudent use of antibiotics
☐ G. Educating healthcare workers, patients, and families
☐ H. Visitor management

11. Which is the preferred hand washing method to prevent transmission of *Clostridium difficile*-associated infections? **SC**

☐ A. Alcohol hand rub
☐ B. Water and soap
☐ C. Iodine solution

12. Which of the following(s) can effectively prevent spreading or protect you from influenza (flu) during the flu season? **MC**

☐ A. Frequent hand washing and avoidance of touching mouth and nose
☐ B. Wearing a mask when you have a flu
☐ C. Covering mouth and nose when sneezing or coughing
☐ D. Staying home and sleeping well
☐ E. Receiving Tamiflu (Oseltamivir) or Relenza (Zanamivir) before catching a flu
☐ F. Receiving Tamiflu (Oseltamivir) or Relenza (Zanamivir) within 36 h of the onset of flu symptoms

13. Which of the followings are the <u>recognized</u> sources of HCAIs? **MC**

☐ A. White coat
☐ B. Nurse uniform
☐ C. Stethoscope
☐ D. Thermometer
☐ E. Wrist watch (used for patient care)
☐ F. Blood pressure cuff (sphygmomanometer)
☐ G. Mattresses and pillows
☐ H. Bedside curtains
☐ I. Chairs/stools/cabinets
☐ J. Air conditioners

14. With regard to HCAIs, visitors may have negative impacts on patients (and community) by: **SC**

☐ A. Disturbing patient's rest
☐ B. Transmitting infectious agents between patients
☐ C. Serving as a carrier of multidrug-resistant pathogens from/to community
☐ D. Disturbing effective infection control measures
☐ E. Smoking within the patients' room

15. With regard to <u>visitor management</u> in hospital settings, which of the following is/are correct? **MC**

☐ A. Visitors should be allowed to stay with patients at any time except during clinical rounds
☐ B. Children visitors should not be allowed to visit patients at all
☐ C. Children visitors should be allowed to visit only their own siblings
☐ D. Visitor screening (for potential infectious diseases) should be observed all time
☐ E. Visitor screening should be observed only during community outbreaks
☐ F. Symptomatic visitors should be excluded unless they have received appropriate medical screening, diagnosis, or treatment

16. Which of the following is/are closely associated with the emergence of <u>multidrug resistant organisms</u> (MDROs)? **MC**

☐ A. Poor infection control guidelines
☐ B. Poor adherence to recommended infection control practices of healthcare workers
☐ C. Poor prescribing behavior of doctors
☐ D. Poor patient compliance
☐ E. Antibiotic abuse or overuse
☐ F. Antibiotic cycling practice

17. Do you consider all patients potentially contagious? YES ☐, NO ☐

18. Which of the followings from patients do you assume to be infectious? **MC**

☐ A. Blood
☐ B. Nasal discharge
☐ C. Saliva
☐ D. Vomitus
☐ E. Feces
☐ F. Urine
☐ G. Sweat
☐ H. Vaginal secretion
☐ I. Non-intact skin (cut, abrasion, eczema)
☐ J. Mucous membranes (oral cavity, eyes)

19. Do you consider all unsterile needles and sharps are contaminated? YES ☐, NO ☐

20. Do you know about nosocomial infection monitoring program in your hospital? YES ☐, NO ☐

21. If YES, which personnel or environmental sources of infection should be included for microbial cultural analysis? **MC**

☐ A. Air
☐ B. Water
☐ C. Disinfectants and antiseptics
☐ D. Inanimate healthcare objects
☐ E. Environmental surfaces
☐ F. Hands of healthcare workers
☐ G. Anterior nares of healthcare workers

22. The proper <u>minimum</u> spacing between beds in multi-patient rooms should be: **MC**

☐ A. 0.6 meter (or 2 feet)
☐ B. 1 meter (or 3 feet)
☐ C. 1.5 meters (or 5 feet)
☐ D. 0.6 meter with a curtain between beds (for infected/colonized patients)
☐ E. 1 meter with a curtain between beds (for infected/colonized patients)
☐ F. 1.5 meters with a curtain between beds (for infected/colonized patients)

23. Do you know how to wash your hands in the six-step hand washing technique? YES ☐, NO ☐

24. With regard to white coats, check the correct answers: **SC**

☐ A. White coat can prevent hospital-associated infections
☐ B. Long-sleeve white coats are more preventative than short-sleeve white coats
☐ C. A disposable gown should be donned over the white coat when performing invasive bedside procedures
☐ D. It is not required to take off the white coat when going to the canteen inside the hospital

--PRACTICE SECTION--

25. How do you usually wash your hands while ON DUTY? **MC**

- ☐ A. Wash with running water
- ☐ B. Wash with running water and bar soap
- ☐ C. Wash with running water and hand-washing liquid
- ☐ D. Wash with running water and skin disinfectant
- ☐ E. Wash with alcohol hand rub

26. When do you wash your hands? **MC**

- ☐ A. Before meals
- ☐ B. Before performing invasive bedside procedures
- ☐ C. Before and after examining patients
- ☐ D. Before and after touching wounds, but not when gloved
- ☐ E. Between two different procedures on different patients
- ☐ F. Between two different procedures on the same patient
- ☐ G. After using the patient-care devices
- ☐ H. After using the computer and desk in the ward
- ☐ I. On joining and completion of duty
- ☐ J. After taking off gloves

27. When do you wear medical utility (non-sterile) gloves? **SC**

- ☐ A. Using the computer, desk, or patient-care equipment in the ward
- ☐ B. Prescribing drugs
- ☐ C. Performing physical examination on patients
- ☐ D. Making clinical rounds
- ☐ E. None of above

28. What type of personal protection equipment (PPE) would **YOU** wear? *Check more than one if applicable.

	Gown	Mask	Goggles	Face shield	Gloves	None
A. Transporting coughing patients in/out of ward#	☐	☐	☐	☐	☐	☐
B. Drawing blood from a patient	☐	☐	☐	☐	☐	☐
C. Irrigating a septic wound	☐	☐	☐	☐	☐	☐
D. Nasogastric suctioning	☐	☐	☐	☐	☐	☐
E. Performing lumber puncture	☐	☐	☐	☐	☐	☐
F. Visiting the neonatal intensive care unit (NICU)	☐	☐	☐	☐	☐	☐
G. Performing physical examination	☐	☐	☐	☐	☐	☐
H. Attending patients with fecal incontinence	☐	☐	☐	☐	☐	☐
I. Delivering a baby of an HIV-positive woman	☐	☐	☐	☐	☐	☐

29. How often do you clean your stethoscope with antiseptic (e.g., 70% alcohol)? **SC**

- ☐ A. After examining each patient
- ☐ B. Daily
- ☐ C. Weekly
- ☐ D. Monthly
- ☐ E. Never

30. How often do you wash your white coat or nurse uniform? **SC**

- ☐ A. Daily
- ☐ B. 3 times a week
- ☐ C. Twice a week

☐ D. Weekly
☐ E. Monthly
☐ F. Never

31. Have you ever been splashed by patient's blood, vomit, or other bodily fluids? YES ☐, NO ☐

32. Have you ever sustained a **used** needlestick injury? YES ☐, NO ☐

33. What action would you take <u>immediately</u> after exposure to HBV- or HIV-contaminated bodily fluid? **MC**

	wash liberally with			followed by
	water	soap	scrubbing	antiseptics
A. Healthy intact skin	☐	☐	☐	☐
B. Wound or non-intact skin (e.g., cut, abrasion, eczema)	☐	☐	☐	☐
C. Mucous membranes (e.g., eyes)	☐	☐	☐	☐

34. What kind of patients would you mask during transfer/transport out of room for investigations? **MC**

☐ A. Patients suffering from influenza
☐ B. Coughing patients with suspected pulmonary tuberculosis
☐ C. Patients receiving radiation therapy for colon cancer
☐ D. All patients regardless of their illness

35. Where should infectious waste from patients be disposed of? **SC**

☐ A. Yellow garbage bag
☐ B. Black garbage bag
☐ C. Red garbage bag
☐ D. No idea

36. Have you ever treated cases of multidrug resistant (MDRO) infection? **PO** YES ☐, NO ☐

37. Your prescribing practice is based on: **SC** & **PO**

☐ A. your own experience
☐ B. the practice of your seniors
☐ C. the hospital/national guidelines
☐ D. the information provided with the drug box
☐ E. Chinese pharmacopoeia

38. What is your selection criterion (the order of importance) when prescribing antibiotics? **PO**
A. Antimicrobial spectrum
B. Cost
C. Brand
D. Generation
Order of importance: 1) _______ 2) _______ 3) _______ 4) _______

39. When do you prescribe/administer antibiotics to patients with an apparent infection? **SC** & **PO**

☐ A. As soon as patients are admitted to the ward
☐ B. After receiving microbiological culture and sensitivity (C &S) results
☐ C. After taking clinical samples for microbiological examination
☐ D. Before taking clinical samples for microbiological examination
☐ E. Before taking clinical samples for microbiological examination but make changes after the C&S results
☐ F. After taking clinical samples for microbiological examination and make changes after the C&S results

40. Your decision to switch antibiotics in the course of anti-infection therapy is based on: **MC** & **PO**

☐ A. Unabated fever
☐ B. Clinical response
☐ C. Culture and sensitivity (C&S) report

☐ D. Patient's affordability
☐ E. Other reasons; please specify:

41. Do you think your hospital has good infection control management and practice? YES ☐, NO ☐

42. If NO, what and how should be improved? Please feel free to write your suggestion. **PO**
